# Foodborne Transmission of Deformed Wing Virus to Ants (*Myrmica rubra*)

**DOI:** 10.3390/insects10110394

**Published:** 2019-11-07

**Authors:** Daniel Schläppi, Patrick Lattrell, Orlando Yañez, Nor Chejanovsky, Peter Neumann

**Affiliations:** 1 Institute of Bee Health, Vetsuisse Faculty, University of Bern, 3097 Bern, Switzerland; patrick.lattrell@students.unibe.ch (P.L.); orlando.yanez@vetsuisse.unibe.ch (O.Y.); ninar@volcani.agri.gov.il (N.C.); peter.neumann@vetsuisse.unibe.ch (P.N.); 2 Department of Entomology, Agricultural Research Organization, the Volcani Center, Bet Dagan 50250, Israel; 3 Swiss Bee Research Centre, Agroscope, 3097 Bern, Switzerland

**Keywords:** *Apis mellifera*, deformed wing virus, foodborne transmission, invasive species, *Myrmica rubra*

## Abstract

Virus host shifts occur frequently, but the whole range of host species and the actual transmission pathways are often poorly understood. Deformed wing virus (DWV), an RNA virus described from honeybees (*Apis mellifera*), has been shown to have a broad host range. Since ants are often scavenging on dead honeybees, foodborne transmission of these viruses may occur. However, the role of the ant *Myrmica rubra* as an alternative host is not known and foodborne transmission to ants has not been experimentally addressed yet. Here, we show with a 16-week feeding experiment that foodborne transmission enables DWV type-A and -B to infect *M. rubra* and that these ants may serve as a virus reservoir. However, the titers of both plus- and minus-sense viral RNA strands decreased over time. Since the ants were fed with highly virus-saturated honeybee pupae, this probably resulted in initial viral peaks, then approaching lower equilibrium titers in infected individuals later. Since DWV infections were also found in untreated field-collected *M. rubra* colonies, our results support the wide host range of DWV and further suggest foodborne transmission as a so far underestimated spread mechanism.

## 1. Introduction

Emerging infectious diseases (EIDs) can cause a significant impact on human- and animal health [1,2,3] and are often the product of a host shift, where a pathogen jumps from its original host to new species [4]. Prominent examples include severe acute respiratory syndrome (SARS) coronavirus or the still ongoing human immunodeficiency virus (HIV-1) pandemic [3,4,5]. Pathogens and their novel hosts lack a co-evolutionary history, which can lead to drastic effects on host populations [6]. In particular, RNA viruses have a high potential to cross species barriers because of high mutation rates that enable fast adaptive changes [7,8], which make them prominent among EIDs [9]. Such RNA viruses have also been suggested to contribute to the recent decline of wild pollinators and losses of managed ones, thereby potentially endangering valuable pollination services [10,11,12,13]. Thus, it is not surprising that virus transmission between managed honeybees, *Apis mellifera*, and wild bees has recently been speculated [14,15].

The transmission potential of honeybee viruses between bees and other ecosystem service providing insects has received considerably less attention [16]. It appears as if cross-species transmission of honeybee viruses frequently happens, although it is not fully understood why and when pathogens jump between some species and not others [4,15].

The deformed wing virus (DWV) is a prevalent virus in honeybees with a broad host range spanning at least eight insect orders and three orders of Arachnida [17,18,19,20,21]. This virus is a positive sense single-stranded RNA virus and a harmful honeybee pathogen [22], of which at least three distinct genotypes or master variants—type A, B, and C—are known [21]. DWV can cause clinical symptoms such as crippled wings, a shortened abdomen, and a reduced host lifespan [22,23,24]. Driven by efficient vector transmission via ectoparasitic mites *Varroa destructor*, DWV has become ubiquitous [25,26,27,28]. Foodborne virus transmission appears to be less important in honeybees, but may be relevant for transmission to other species [21], especially for predators and scavengers [29,30,31,32]. However, data on foodborne DWV transmission between honeybees and insect predators/scavengers are scarce.

Since virus-infected brood and adults are often expelled from honeybee colonies [33,34,35,36] and ants are often seen foraging such bees in apiaries (personal observations), it appears as if ants are very likely to consume DWV-infected food. However, there are only few studies on virus transmission between honeybees and ants. DWV and Kashmir Bee Virus (KBV) presence plus replication was detected in invasive argentine ants (*Linepithema humile*) as well as presence of Black queen cell virus (BQCV) in *Linepithema humile* and *Anoplolepis gracilipes* [37,38,39]. Further, DWV and other honeybee viruses (BQCV, Israeli acute paralysis virus and Sacbrood virus) were detected in *Camponotus* sp. ants [19]. Similarly, chronic bee paralysis virus seems to be able to replicate in ants *Camponotus vagus* [29]. So far, no study has yet experimentally addressed the efficacy of foodborne virus transmission between honeybees and predators/scavengers. Since ants are ubiquitous and play essential roles in terrestrial ecosystem functioning [40,41,42,43], virus transmission from managed honeybees might pose a considerable threat, given the virus is able to exploit the novel hosts. On the other hand, viruses and other pathogens may help limiting the spread of invasive *M. rubra* populations [37,44]. Novel hosts can be biological vectors that support virus replication or reservoirs and mechanical vectors without virus replication [4]. Further, spillover events can also be bidirectional, from the novel host back to the initial host [1], possibly resulting in altered virulence and respective consequences as e.g., for influenza [45].

Here, we empirically test for the first time whether ants, *Myrmica rubra*, which are common in Europe [46] and an invasive species in North America [47], can be alternative hosts of DWV and if foodborne transmission is feasible. Monitoring of viral loads after consumption of virus-infected food will shed light on the fate of the viruses. If negative-sense strands of the RNA viruses can be found, this would suggest virus replication [48,49].

## 2. Materials and Methods

### 2.1. Experimental Set-up

Fifteen *M. rubra* colonies, collected in their founding stage around Berlin, Germany, were purchased from https://www.antstore.net/shop/de [50]. These colonies were kept in nesting tubes (155 mm length, 14 inner diameters) and fed with *Drosophila hydei* and sugar-water *ad libitum* (40% mass fraction of sugar). For the experiment, the colonies were transferred into boxes (60 × 70 × 15 mm) attached to a foraging arena (200 × 150 × 120 mm). Humidity in the nest was maintained with a moist cotton ball. Red foil covered the nest boxes to reduce stress [51]. The foraging arenas had an opening covered with removable insect nets and the edges were covered with paraffin oil to minimize escape. The colonies were maintained at RT (19–23 °C) with a natural day/night cycle.

### 2.2. Preparation of DWV-Infected Honeybee Pupae

White-eyed honeybee pupae were obtained from sealed worker brood frames of two local *A. mellifera* colonies and then microinjected laterally between the second and third segment of the abdomen with 2 μL of a solution containing DWV [52]. The pupae were then incubated at 34.5 °C, ≥50% relative humidity and darkness for seven days [53] and afterward frozen at −80 °C. The DWV solution was prepared by homogenizing a DWV symptomatic honeybee in PBS and chloroform, followed by centrifugation (15800 rcf for 10 min). The supernatant was collected, diluted 1:1000, and frozen at −80 °C before injection into the pupae [52]. For the injected honeybee pupae used as food and positive controls for the virus detection, the logarithmic mean number of viral copies was 11.49 ± 0.29 and DWV type-specific primers showed a ratio of 5.88/1 for DWV-B to DWV-A, but no DWV-C.

### 2.3. Feeding Regime

After four weeks, the colonies were randomly assigned to three treatments, differing in DWV infected protein supply over eight-weeks: (1) The controls (C, N = 3) were fed with *D. hydei ad libitum*; (2) treatment 1 (T1) colonies (N = 6) were fed for four weeks with DWV-infected honeybee pupae and then for eight weeks with *D. hydei*; (3) treatment 2 (T2) colonies (N = 6) were fed for eight weeks with DWV infected honeybee pupae and for 4 weeks with *D. hydei. D. hydei* were chosen as food for the controls, because honeybee pupae taken from local Swiss colonies inevitably contain background levels of DWV [54]. Sugar water (40% mass fraction of sugar) supply *ad libitum* was maintained. Then, ten adult workers were randomly sampled from each colony over four consecutive weeks until the end of the experiment (week 13–16) and stored at −80 °C until further processing. The different feeding regimes were chosen to see if there is an impact of feeding duration on virus uptake and the time gap since the last feeding of the virus until the first sampling should reduce the risk of detecting DWV in the ant’s gut content [55,56]. Some colonies were not large enough to provide adults, thereby decreasing sample size at the last sampling (C – N = 1; T1 – N = 5; T2 – N = 6).

### 2.4. RNA Extraction

For the quantification of viral titers a pooled sample of five workers per sampling week was crushed in 100 µL TN buffer (100 mM Tris, 100 mM NaCl, pH 7.6) with a 5-mm metal bead in 2-mL Eppendorf^®^ (Basel, Switzerland) tubes using a Retsch^®^ (Haan, Germany) MM 300 mixer mill for 1 min at the frequency 25 1/s [57]. Injected honeybee pupae extracted as well as positive controls using 200 µL TN buffer. Fifty microliters of the homogenate was transferred to 1.5-mL Eppendorf^®^ tubes and the RNA extraction was done using a NucleoSpin^®^ RNA II kit (Macherey-Nagel, Oensingen, Switzerland) following the manufacturer’s recommendations. In the final step, RNA was eluted in 30 µL of elution buffer and stored at −80 °C [57]. To monitor the efficiency of the RNA purification and cDNA synthesis, tobacco mosaic virus (TMV, 1 μL TMV solution (1:1000)) was used as an exogenous internal reference added to each sample at the first step of the RNA extraction [58].

### 2.5. Reverse Transcription

For the reverse transcription, we used the M-MLV RT (Promega, Dübendorf, Switzerland) Kit and followed the manufacturer’s recommendations: Template RNA (50–500 μg, Table S1) was incubated with 0.75 μL of a random hexamer oligonucleotide (100 μM) and H_2_O for 5 min at 70 °C in a thermocycler (Biometra; Analytik Jena, Jena, Germany). For the cDNA synthesis, 5 μL 5× buffer, 1.125 μL nucleoside triphosphate (dNTP) 10 mM and 1 μL reverse transcriptase (M-MLV) was added to a final reaction volume of 25 μL, which was incubated at 37 °C for 60 min. The resulting cDNA was diluted 1/5 and stored at −25 °C until further processing.

### 2.6. Quantitative PCR

The RT-qPCR was performed in duplicate for each sample using the KAPA SYBR^®^ FAST Universal qPCR kit (KAPA Biosystems, Wilmington, DE, USA) with 12 μL volumes containing 6 μL KAPA SYBR^®^ (Wilmington, DE, USA) green reaction mix, 0.24 μL each of the forward and reverse primers for DWV or TMV (Table 1), 2.52 μL H_2_O and 3 μL diluted cDNA [52]. An ECO™ real-time PCR machine (Illumina, San Diego, CA, USA) processed the reaction at the following qPCR cycling profile: 3 min incubation at 95 °C and 40 cycles of 3 s at 95 °C for denaturation, 30 s at 57 °C for annealing and extension, and data collection. By reading the fluorescence at 0.5 °C intervals between 55 °C and 95 °C, a melting curve analysis was performed after the amplification to verify the specificity of the PCR products. On each plate, a ten-folds serial dilution of purified PCR products that served as standard curves for DWV and TMV and two no-template negatives were included [59]. From the q-PCR output data, the standard curves and the experimental dilution factors viral titers respectively estimated viral copies per sample were derived [60]. To account for the exponential distribution of the data, virus titers were log-transformed and throughout the manuscript, the logarithmic values of the viral titers are reported. Because it is not possible to log transform zero values of negative samples we assigned to them a hypothetical Cq value of 36, which was transformed to virus titers as above. From the averaged value of these “negative titers,” we obtained the titer detection threshold [61].

### 2.7. DWV Negative-Sense Strand Analyses

The detection of the negative-sense strand RNA was used as a token of DWV replication [48,49]. First, the negative-sense strand RNA was tagged and then amplified: The cDNA of all T1 and T2 colonies sampled at Week 13 and 16 were obtained by using Superscript^®^ III reverse transcriptase (Invitrogen, Carlsbad, CA, USA) following the manufacturer’s recommendations (1 μL of DWV 3F tagged primer, Table 1, 1 μL dNTP 10 mM, 4 μL of 5× first strand buffer, 2 μL 0.1 M DTT, 1 μL M-MLV reverse transcriptase, H_2_O and sample in a final reaction volume of 20 μL) [52]. The reaction was processed in a thermocycler (Biometra; Analytik Jena, Jena, Germany) according to the following protocol: 5 min at 65 °C, 10 min at 25 °C, 60 min at 50 °C followed by 15 min at 70 °C. The obtained cDNA then was purified using the NucleoSpin^®^ Gel & PCR Clean-up kit (Macherey-Nagel) and eluted in 30 μL elution buffer to avoid false-positive results. A five-fold dilution of the purified cDNA was used for the subsequent amplification with conventional PCR (MyTay™ kit by Bioline, London, UK). DWV-R1 was used as the reverse primer, while a Tag oligonucleotide (Table 1) was used as forward primer. For each sample, a second reaction was carried out without forward primer to check for efficient removal of DWV 3F tagged primer after the reverse transcription. The thermal cycling was done with a profile that consisted of 2 min incubation at 95 °C and 35 cycles of 20 s at 95 °C for denaturation, 20 s at 42 °C for annealing, and 30 s at 72 °C for extension. By electrophoresis on a 1.5% agarose gel, the PCR products were analyzed and visually checked under UV light. If a clear band was present at 221 bp and the associated negative control was indeed negative, a sample was considered positive.

### 2.8. Statistical Analyses

All statistical analyses were performed using R version 3.5.1 [64] and R Studio version 1.1.456 [65]. Data were checked for normality with the Shapiro-Wilk test and homogeneity of variances with the Levene’s test and subsequent statistical tests chosen accordingly. We used the lmer function from the package lme4 [66] to perform a linear mixed effect analysis to test for differences of viral titers over time between the treatments. The logarithm of the viral titers entered the model as a response variable. As fixed effects, we used sampling week and treatment including their interaction term. As a random effect, we added the colony identity to control for multiple testing of the same colonies (week 13–16). Visual inspection of residual plots did not reveal deviations from homoscedasticity or normality. *p*-values were obtained by comparing the model with the effect in question against the model without it, using an ANOVA as a likelihood ratio test. The post-hoc pairwise comparison was then performed using the Bonferroni correction.

## 3. Results

### 3.1. DWV Genomic Copies

**Figure 1:**
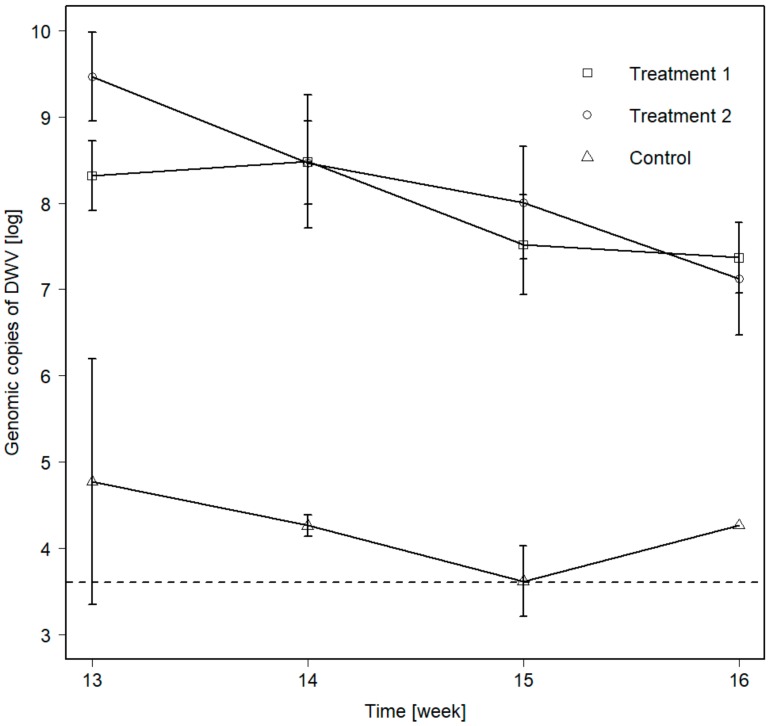
Genomic copies of DWV-A&B in *Myrmica rubra* workers under different virus feeding regimes (Control = 0 weeks, Treatment 1 = 4 weeks, Treatment 2 = 8 weeks). Means and standard deviations, as well as the detection threshold [59] are shown.

All the samples from T1 (colonies fed for four weeks with DWV-infected honeybee pupae) and T2 (colonies fed for eight weeks with DWV-infected honeybee pupae) were strongly positive, with two exceptions (one pooled sample each during week 13 and 15). With 4.17/1 (T1) and 5.46/1 (T2), the ratio of DWV type-B to type-A was similar to the one in the feeding pupae (5.88/1 for DWV-B to DWV-A). Sequencing of the PCR products confirmed the identity of both DWV strains found in *M. rubra* (GenBank accession: DWV-A, JF346565.1, 99.73% identity, 100% query cover; DWV-B, AY251269.2, 98.84% identity, 99% query cover). The viral titers found in ants were in the range of 6.46–9.66 with a log mean of 7.95 ± 0.72 for T1, 6.17–10.18, 8.26 ± 1.02 for T2, and 3.17–5.88, 4.22 ± 0.85 in the control group seen over all weeks (Figure 1, Table 2). Viral titers in ants were significantly affected by the factors treatment (*X*^2^_(2)_ = 42.39, *p* < 0.001) and sampling week (*X*^2^_(1)_ = 38.43, *p* < 0.001), including their interaction term (*X*^2^_(2)_ = 8.12, *p* < 0.017). Viral titers of both treatment groups were significantly higher than in the control group (*p* < 0.0001, Bonferroni post hoc test), but there was no significant difference between the two treatment groups (*p* = 0.53, Bonferroni post hoc test). The significant interaction between treatment and sampling week indicates that the slope of the viral titer decrease was dependent on treatment.

### 3.2. Negative-Sense Strand Specific PCR

**Figure 2:**
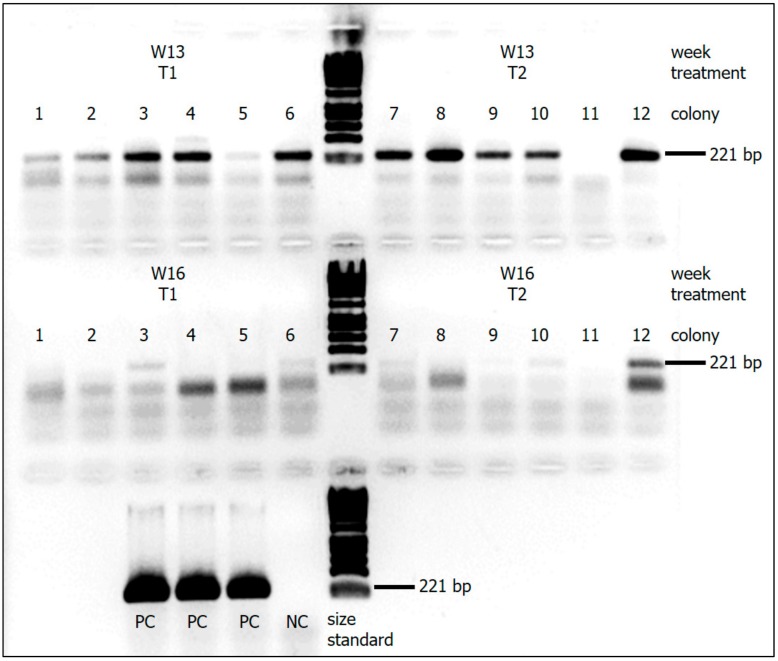
DWV-negative-sense strand detection in ants *Myrmica rubra*. For both treatments T1 and T2 samples are shown 13 weeks (W13) and 16 weeks (W16) after start of the feeding experiment. Each lane represents a pooled sample of five workers of the respective colony (C1–C12). Presence of a band at 221 bp shows the negative-sense strand and thus indicates virus replication (NC = negative control (distilled water); PC = positive control (DWV-injected honeybee pupae)).

The presence of the negative-sense strand of DWV, as indicated by a band at 221 bp of the agarose gel electrophoresis [49,52] was confirmed for all colonies of T1 at week 13, but only for two colonies at week 16 (Figure 2). Similarly, the band at 221 bp was clearly visible in all but one colony of T2 at the first sampling point. Four weeks later, the band was still well visible in one colony, less pronounced in two others, and not visible in three colonies.

## 4. Discussion

Our results suggest that the ant *M. rubra* can be an alternative host for both DWV A and B as a result of foodborne transmission because of the presence of both high viral titers and the negative-sense RNA strand for up to 13 weeks after the last virus-infected food has been consumed. These results further support that DWV has a wide host range including other predatory insects [14,15,17,18,19,20,21,29,30,31,32,37]. Interestingly, there were only two ant species from the family Formicidae among the more than 60 species in which DWV has been detected so far [21]. It is obvious that recently consumed highly virus infected food like honeybee pupae, containing the virus negative-sense strand, might lead to false positive results simply because of the presence of viral particles in ant’s gut. Because of the delayed sampling, we can however expect that the gut content of the ants has been cleared out at least once before the virus analyses [55].

The experimental data show that foodborne transmission, e.g., via consumption of infected honeybees, can be an underlying mechanism, which enables DWV to infect *M. rubra* and potentially other arthropod predators and scavengers [29,30,31,32,37]. Indeed, both treatment groups that were fed with virus-infected honeybee pupae had consistently higher viral titers than the controls. Since symptomatic honeybees with an overt infection have 10^10+^ genomic equivalents of DWV and at lower infection levels 10^3^–10^9^ genomic copies [67], the titers observed in *M. rubra* seem to reflect covert infections. However, the average honeybee worker (~120 mg) weights about 60 times more than a *M. rubra* worker [68,69]. When controlled for these bodyweight differences, the DWV A and B titers found in the treatment groups therefore almost reach infection levels per mg ant-tissue analogous to an overt infection in bees. Further, the viral titers were very low in the control colonies, thereby indicating that DWV A and B were only replicating in the treatments. Interestingly, the ratios of DWV type-B to type-A were very similar in the ants, regardless of the treatment (4.17/1 (T1), 5.46/1 (T2), as well as in the injected honeybee pupae (5.88/1 for DWV-B to DWV-A), thereby suggesting that both strains had similar reproductive capacities in both hosts.

Over time, a decrease of both the viral titers and the presence of the negative-sense strand was observed in the treatment colonies. It may well be that the treatment colonies would eventually reach a low viral level such as the controls, display low level replication and at that both A and B strains are present. However, we have no data to address those questions. What could explain the observed decrease of viral titers over time? Neither altruistic self-removal of infected nestmates [70] nor increased mortality in the treatment colonies were observed. Therefore, the higher initial viral titers and the more common minus-strand presence could reflect virus particles consumed with the infected honeybee food. In light of ant physiology [71], this is nevertheless unlikely to explain the comparatively high viral titers and especially the presence of the minus strand for up to 13 weeks after the last virus-infected food has been consumed. The most parsimonious explanation therefore is that the ants were fed with highly virus saturated honeybee pupae resulting in initial viral peaks, then approaching lower equilibrium titers in infected ant individuals later. In light of these results, it appears as if controlled laboratory experiments are required before drawing general conclusions on the role of predators/scavengers as alternative hosts of any honeybee viruses, i.e., the timing of the last virus-infected food consumption should be known. Similarly, the detection of the negative-sense strand in *V. destructor* suggested DWV replication in the mites until recently, when a non-propagative manner DWV transmission has been proposed [72].

Given the prevalence and high titers of DWV and its wide host range [21], it is not surprising that the field-collected control colonies were also tested positive for DWV at low levels. Since our experimental data clearly show that DWV transmission to ants is feasible via food, we assume that either (1) DWV-contaminated food must have been given to the ant colonies in captivity prior to our experiments or alternatively, but not mutually exclusive, (2) the *D. hydei* flies used as control food were DWV positive, which we regard as unlikely, or (3) the control colonies had consumed DWV-infected food prior to field-sampling by the company. Given the latter case, this would support the view that DWV transmission to *M. rubra* also occurs in the field, which would be in line with other studies showing the presence of viruses, originally described from honeybees, in ants [29,37]. A closer look at the controls also reveals that the viruses are not equally distributed within these naturally infected colonies. It would be interesting to see if the uneven distribution is due to age-related division of labor and non-stochastic interaction patterns [73], i.e., foragers might have a higher exposure risk compared to in-house workers. Considering the low viral titers in the controls, it is possible that the viruses persist in *M. rubra* as a covert infection that might turn into an overt infection under suitable conditions, e.g., parasite infections as in honeybees [26].

Although the data suggest that DWV prevails and replicates in *M. rubra* and that it also occurs in field-collected samples, it provides no insights on pathogenicity. Neither abnormalities in colony development nor individual clinical symptoms in workers were observed. Additional studies are therefore required to understand if and how honeybee viruses might affect *M. rubra* or other predators/scavengers.

## 5. Conclusions

The data suggest for the first time that *M. rubra* is another biological host for DWV type-A and -B known from honeybees and that foodborne transmission is an underlying transmission mechanism. Since ants are ubiquitous and important in terrestrial ecosystems, it appears as if there is a considerable potential for virus transmission between wild insects and managed bees with possible consequences for ecosystem functioning. It has been suggested that viruses might offer novel options for the control of invasive ant species [37]. However, we consider such biocontrol suboptimal due to the evident wide host range of viruses and the here demonstrated considerable chances of host-switches, with possibly dramatic consequences [5].

## Figures and Tables

**Table 1:** Primers used during qPCR for the detection and quantification of deformed wing virus (DWV) and minus strand-specific PCR as a proxy for viral replication as well as the DWV type-specific (A, B, and C) primers.

Target	Primer	Sequence (5′–3′)	Size [bp]	Reference
DWV	DWV-F8668	TTCATTAAAGCCACCTGGAACATC	136	[60]
	DWV-B8757	TTTCCTCATTAACTGTGTCGTTGA	136	
DWV	DWVnew-F1	TACTAGTGCTGGTTTTCCTTT		[62]
DWV-A	DWVA-R1	CTCATTAACTGTGTCGTTGAT	155	[62]
DWV-B	DWVB-R1	CTCATTAACTGAGTTGTTGTC	155	[62]
DWV-C	DWVC-R1	ATAAGTTGCGTGGTTGAC	152	[62]
TMV	TMVQ1-fwd	TGTAGCGCAATGGCGTACAC	55	[58]
	TMVQ1-rev	CATGCGAACATCAGCC AATG	55	
DWV minus-strand	DWV 3F-Tag	AGCCTGCGCACCGTGG–GGATGTTATCTCCTGCGTGGAA	221	[63]
	Tag	AGCCTGCGCACCGTGG	221	[49]
	DWV4-R1	TGTCGAAACGGTATGGTAAACT	221	This study

**Table 2:** Genomic copies of DWV-A and B in *Myrmica rubra* workers. Logarithmic means ± standard deviations are shown over the four weeks of sampling. Sample sizes are given in brackets for each week.

	Viral Titer (Log Mean ± sd)			
Treatment	Week 13	Week 14	Week 15	Week 16
Control	4.77 ± 1.42 [3]	4.26 ± 0.12 [3]	3.44 ± 0.29 [3]	4.27 [1]
Treatment 1	8.32 ± 0.41 [6]	8.48 ± 0.77 [6]	7.52 ± 0.58 [6]	7.37 ± 0.41 [5]
Treatment 2	9.47 ± 0.51 [6]	8.47 ± 0.48 [6]	8.01 ± 0.65 [6]	7.12 ± 0.65 [6]

## Supplementary Materials

The following are available online at https://www.mdpi.com/2075-4450/10/11/394/s1, Table S1: DWV titers and positive/negative assignment of colonies will be available online.
