# Supplementary material for: Foodborne Transmission of Deformed Wing Virus to Ants (Myrmica rubra)
Source: Insects. 2019 Nov 7;10(11):394. doi: 10.3390/insects10110394 (PMC6920936; doi:10.3390/insects10110394)
Supplement: Supplementary file 1 [file insects-10-00394-s001.pdf]

insects

Article

Foodborne Transmission of Deformed Wing Virus to Ants (*Myrmica rubra*)

Daniel Schläppi [1,*], Patrick Lattrell [1], Orlando Yañez [1], Nor Chejanovsky [1,2] and Peter Neumann [1,3]

1 Institute of Bee Health, Vetsuisse Faculty, University of Bern, 3097 Bern, Switzerland; patrick.lattrell@students.unibe.ch (P.L.); orlando.yanez@vetsuisse.unibe.ch (O.Y.); ninar@volcani.agri.gov.il (N.C.); peter.neumann@vetsuisse.unibe.ch (P.N.)
2 Department of Entomology, Agricultural Research Organization, the Volcani Center, Bet Dagan 50250, Israel
3 Swiss Bee Research Centre, Agroscope, 3097 Bern, Switzerland
* Correspondence: daniel.schlaeppi@vetsuisse.unibe.ch; Tel.: +41-31-631-57-69

Received: 30 August 2019; Accepted: 5 November 2019; Published: date

Table S1. Experimental data with genomic copies of DWV-A&B per individual *Myrmica rubra* worker and *Apis mellifera* pupae used for feeding the ants. All samples from all treatments (1 = Treatment 1; 2 = Treatment 2; 3 = Control; 4 = Honeybee pupae) and sampling weeks (13–16) are reported including the amount of template RNA used for reverse the transcription.

Sample_Name	Treatment *	Sampling week	Positive/Negative Assignment	Genomic DWV Copies per Individual	Template RNA
T1_1	1	13	positive	6.04×10^7	500
T1_2	1	13	positive	4.94×10^8	250
T1_3	1	13	positive	7.87×10^7	100
T1_4	1	13	positive	4.71×10^8	250
T1_5	1	13	positive	1.78×10^8	50
T1_6	1	13	positive	4.07×10^8	50
T2_1	2	13	positive	6.12×10^9	50
T2_2	2	13	positive	2.35×10^9	250
T2_3	2	13	positive	4.69×10^9	50
T2_4	2	13	positive	8.99×10^8	50
T2_5	2	13	positive	6.92×10^8	50
T2_6	2	13	positive	1.50×10^{10}	50
C_1	3	13	positive	1.85×10^5	100
C_2	3	13	negative	1.48×10^3	250
C_3	3	13	positive	7.58×10^5	500
2_T1_1	1	14	positive	1.11×10^8	500
2_T1_2	1	14	positive	4.66×10^9	250
2_T1_3	1	14	positive	7.58×10^8	250
2_T1_4	1	14	positive	6.82×10^7	250
2_T1_5	1	14	positive	6.28×10^8	250
2_T1_6	1	14	positive	4.43×10^7	250
2_T2_1	2	14	positive	1.65×10^8	250
2_T2_2	2	14	positive	2.89×10^8	250
2_T2_3	2	14	positive	1.02×10^9	250
2_T2_4	2	14	positive	9.80×10^7	250
2_T2_5	2	14	positive	1.06×10^8	250
2_T2_6	2	14	positive	1.22×10^9	250
2_C_1	3	14	positive	2.00×10^4	250
2_C_2	3	14	positive	1.34×10^4	250
2_C_3	3	14	positive	2.32×10^4	250
3_T1_1	1	15	positive	7.87×10^7	50
3_T1_2	1	15	positive	1.77×10^7	250

3_T1_3	1	15	positive	5.22×10^{7}	50
3_T1_4	1	15	positive	9.53×10^{7}	100
3_T1_5	1	15	positive	2.88×10^{6}	50
3_T1_6	1	15	positive	6.51×10^{7}	250
3_T2_1	2	15	positive	1.08×10^{8}	250
3_T2_2	2	15	positive	8.90×10^{7}	50
3_T2_3	2	15	positive	2.07×10^{8}	50
3_T2_4	2	15	positive	2.50×10^{8}	250
3_T2_5	2	15	positive	5.85×10^{6}	250
3_T2_6	2	15	positive	3.71×10^{8}	50
3_C_1	3	15	positive	2.55×10^{3}	250
3_C_2	3	15	positive	2.27×10^{3}	250
3_C_3	3	15	positive	1.23×10^{4}	50
4_T1_1	1	16	positive	1.93×10^{7}	50
4_T1_3	1	16	positive	1.45×10^{7}	50
4_T1_4	1	16	positive	4.93×10^{7}	100
4_T1_5	1	16	positive	6.98×10^{6}	250
4_T1_6	1	16	positive	7.22×10^{7}	50
4_T2_1	2	16	positive	2.50×10^{7}	50
4_T2_2	2	16	positive	1.77×10^{7}	100
4_T2_3	2	16	positive	6.01×10^{6}	50
4_T2_4	2	16	positive	1.04×10^{7}	50
4_T2_5	2	16	positive	1.50×10^{6}	50
4_T2_6	2	16	positive	1.32×10^{8}	250
4_C_3	3	16	positive	1.84×10^{4}	100
FP_2.1	4	NA	positive	2.10×10^{11}	500
FP_2.2	4	NA	positive	1.66×10^{11}	500
FP_2.3	4	NA	positive	1.18×10^{11}	500
FP_2.4	4	NA	positive	2.98×10^{11}	500
FP_3.1	4	NA	positive	3.17×10^{11}	500
FP_3.2	4	NA	positive	2.54×10^{11}	500
FP_3.3	4	NA	positive	2.18×10^{11}	500
FP_4.1	4	NA	positive	2.44×10^{11}	500
FP_4.2	4	NA	positive	5.81×10^{11}	500
FP_4.3	4	NA	positive	1.12×10^{12}	500
FP_4.5	4	NA	positive	7.23×10^{11}	500
